# Predictive Role of Cluster Bean (*Cyamopsis tetragonoloba*) Derived miRNAs in Human and Cattle Health

**DOI:** 10.3390/genes15040448

**Published:** 2024-04-01

**Authors:** Sarika Sahu, Atmakuri Ramakrishna Rao, Tanmaya Kumar Sahu, Jaya Pandey, Shivangi Varshney, Archna Kumar, Kishor Gaikwad

**Affiliations:** 1 Indian Agricultural Statistics Research Institute, ICAR, New Delhi 110012, India; sarika.sahu@icar.gov.in (S.S.); jayapandey.2552@gmail.com (J.P.); shivangivarshney@live.com (S.V.); 2 Amity Institute of Biotechnology, Amity University, Noida 201303, India; akumar21@amity.edu; 3 Indian Council of Agricultural Research, New Delhi 110001, India; 4 Indian Grassland and Fodder Research Institute, ICAR, Jhansi 284003, India; tanmaya.sahu@icar.gov.in; 5 National Institute for Plant Biotechnology, ICAR, New Delhi 110012, India; kish2012@gmail.com

**Keywords:** plant-derived miRNAs, gene–disease association, cross-kingdom analysis, cattle diseases, human health

## Abstract

MicroRNAs (miRNAs) are small non-coding conserved molecules with lengths varying between 18-25nt. Plants miRNAs are very stable, and probably they might have been transferred across kingdoms via food intake. Such miRNAs are also called exogenous miRNAs, which regulate the gene expression in host organisms. The miRNAs present in the cluster bean, a drought tolerant legume crop having high commercial value, might have also played a regulatory role for the genes involved in nutrients synthesis or disease pathways in animals including humans due to dietary intake of plant parts of cluster beans. However, the predictive role of miRNAs of cluster beans for gene–disease association across kingdoms such as cattle and humans are not yet fully explored. Thus, the aim of the present study is to (i) find out the cluster bean miRNAs (cb-miRs) functionally similar to miRNAs of cattle and humans and predict their target genes’ involvement in the occurrence of complex diseases, and (ii) identify the role of cb-miRs that are functionally non-similar to the miRNAs of cattle and humans and predict their targeted genes’ association with complex diseases in host systems. Here, we predicted a total of 33 and 15 functionally similar cb-miRs (fs-cb-miRs) to human and cattle miRNAs, respectively. Further, Kyoto Encyclopedia of Genes and Genomes (KEGG) analysis revealed the participation of targeted genes of fs-cb-miRs in 24 and 12 different pathways in humans and cattle, respectively. Few targeted genes in humans like LCP2, GABRA6, and MYH14 were predicted to be associated with disease pathways of Yesinia infection (hsa05135), neuroactive ligand-receptor interaction (hsa04080), and pathogenic Escherichia coli infection (hsa05130), respectively. However, targeted genes of fs-cb-miRs in humans like KLHL20, TNS1, and PAPD4 are associated with Alzheimer’s, malignant tumor of the breast, and hepatitis C virus infection disease, respectively. Similarly, in cattle, targeted genes like ATG2B and DHRS11 of fs-cb-miRs participate in the pathways of Huntington disease and steroid biosynthesis, respectively. Additionally, the targeted genes like SURF4 and EDME2 of fs-cb-miRs are associated with mastitis and bovine osteoporosis, respectively. We also found a few cb-miRs that do not have functional similarity with human and cattle miRNAs but are found to target the genes in the host organisms and as well being associated with human and cattle diseases. Interestingly, a few genes such as NRM, PTPRE and SUZ12 were observed to be associated with Rheumatoid Arthritis, Asthma and Endometrial Stromal Sarcoma diseases, respectively, in humans and genes like SCNN1B associated with renal disease in cattle.

## 1. Introduction

In the ecosystem, quite often organisms interact with each other directly or indirectly. The prokaryotic cells communicate through quorum sensing whereas eukaryotic cells transmit signals through hormones and cytokines [1]. In living organisms, quite often microRNAs (miRNAs) regulate multiple cell activities such as stress tolerance, plant defense mechanisms, growth and development [2]. In recent years, it has been reported that miRNAs might have been transmitted from one species to another [3] and might have targeted the genes not only at the endogenous level but also at the exogenous level [4,5]. Moreover, the cross-kingdom mobility of miRNAs has been studied between bacteria and animals [6], plants and insects [7], and plants and fungi [8]. Also, numerous exogenous plant miRNAs exhibit perfect complementarity to human genes as well as to the genes of other mammals [9]. The experimental detection of plant miRNAs in human plasma, serum, urine, saliva and other body fluids have been reported in the recent past [10,11]. The RT-PCR technique reveals the stability of plant miRNAs in the human sera [12,13] and such stability is achieved by the methylation at 2′-hydroxyl group of sugar at 3′ end of miRNA [14]. The presence of plant miRNAs in humans has been reported when plant products in the form of diet travelled through the gastrointestinal tract [15]. It was reported earlier that plant miRNA “MIR168a” targets the low-density lipoprotein receptor (LDL) adaptor protein 1 (*LDLRAP1*) of humans’ liver cells that led to the uptake of low-density lipoprotein receptor from blood [3]. Additionally, it was showed that human miRNAs might have translocated and regulated the genes responsible for growth of *Plasmodium falciparum* [16]. The studies on virus–host interaction showed that viruses used host miRNA biosynthesis machinery for the expression of their own miRNAs [17]. In addition, the oilve miRNAs, viz., oeu-sR20, oeu-sR27 and oeu-sR34 showed functional homology with human miRNA “hsa-miR34” that regulates the expression of genes in human tumor cells [15]. Recently, it was found that miRNAs of Avacado (*Persea americana*) regulate the function of human genes like *FLT1* (Fms Related Tyrosine Kinase 1) and *SOCS3* (Suppressor of Cytokine Signalling 3) [10]. The miR160 and miR2673 of *Brassica oleracea* reported to regulate the expression of human lung-cancer-related genes [18].

The cluster bean, also known as guar, is a drought-tolerant crop of the legume family. It is grown in India for vegetable, green manure and seed production. It is also recognized as a medicinal plant and possesses a high quantity of phytochemicals [19,20]. It is commonly used to cure various diseases like ulcer, secretion, hyperglycemia, and cathartic [21]. The edible parts of this crop are consumed by humans and cattle as food and fodder. Through such intake, probably the cluster bean plant-derived miRNAs might have been translocated to humans and cattle. Subsequently, these miRNAs might have played different roles in the regulation of gene–disease association in animals. Thus, in the present study, we tried to identify the cluster bean miRNAs (cb-miRs) that are functionally similar and functionally non-similar to miRNAs of humans and cattle. Further, we predicted the target genes of the identified functionally similar cb-miRs (fs-cb-miRs) and functionally non-similar cb-miRs (fns-cb-miRs), pathways involving the predicted genes, and the gene–disease associations. Thus, the present findings may supplement the existing knowledge on the role of cb-miRs in regulating genes associated with human and cattle diseases. Additionally, the findings may help promote the use of cluster bean plant parts as dietary supplement for humans and as fodder for animals. The findings may act as a remedial outbreak against various diseases as well as in advising therapeutic strategies for animal diseases.

## 2. Material and Methods

### 2.1. Data Source

A total of 171 and 21 cluster bean miRNAs were collected from [22] and [23], respectively, after removing the redundancy. The prefixes Ct and Cte of cluster bean miRNAs, used earlier [22,23], were retained as such in their ids. For functional-similarity study of cb-miRs, a total of 1052 miRNAs of cattle and 2781 miRNAs of human were collected from miRbase [24]. The mRNA sequences of cattle were downloaded from the National Center for Biotechnology Information (NCBI), whereas human mRNAs available in the database of psRNATarget server [25] were considered in the study. The miRNAs and mRNAs of cattle and humans were used to carry out the cross-kingdom analysis.

### 2.2. Cross-Kingdom miRNA Similarity

**Figure 1:**
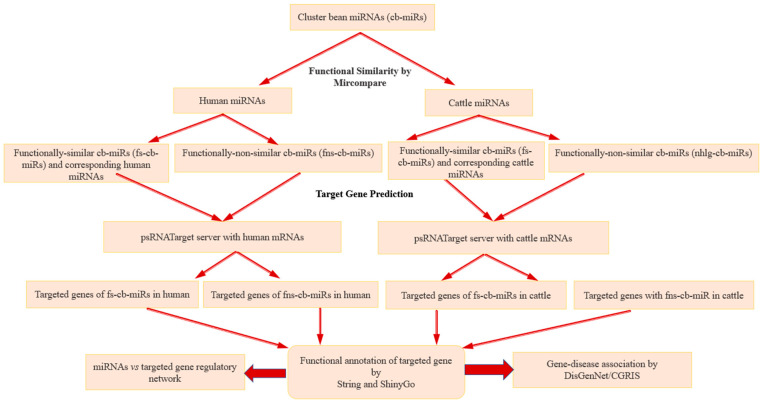
Workflow of cross-kingdom gene regulation through miRNAs of cluster beans in humans and cattle.

The cross-kingdom mechanisms involve cell communication as well as intra-species and inter-species interactions through miRNAs. Here, a total of 192 cb-miRs (=171 + 21) were considered to perform cross-kingdom analysis in human and cattle. The MirCompare tool [26] was used to predict the similarity between cb-miRs and human miRNAs, cattle miRNAs with suitable parameters (r-value 0.55, similarity ≥ 60% and seed-region threshold value 5). The cb-miRs with the said remarkable and high similarity were considered as s-cb-miRs. Further, the secondary structures of pre-miRNAs of cluster beans, humans and cattle were predicted by RNA module of Vienna Package to filter out those s-miRNAs satisfying the structural properties from the miRNAs identified by MirCompare, i.e., those s-miRNAs (mature miRNAs of length 21 nt) localized on the secondary structure of pre-miRNAs. While s-cb-miRs that have sequence homology < 60% with miRNAs of cattle and human were considered as non-similar cb-miRs (ns-cb-miRs). Thus, the total miRNAs of cluster bean were bifurcated into s-cb-miRs and ns-cb-miRs. The s-cb-miRs may have functional and likely evolutionary importance while the ns-cb-miRs may have functional importance alone. Therefore, both types of cb-miRs were studied for the regulation of genes and their associated diseases in humans and cattle. The workflow meant for cross-kingdom analysis of miRNAs is given in Figure 1.

### 2.3. Identification of Functionally Similar cbmiRs (fs-cb-miRs) and Prediction of Potential Target Genes in Humans and Cattle

The s-cb-miRs and their corresponding paired miRNAs in humans and cattle were considered for target gene prediction and, thus, submitted in the psRNATarget server [25]. Subsequently, only those genes that are commonly targeted by the referred pairs (s-cb-miRs and corresponding paired miRNA in the host system) are considered and referred as “targeted genes” for further down-stream analysis on annotation, pathways and gene–disease association whereas the s-cb-miRs in the referred pairs are referred as functionally similar cb-miRs (fs-cb-miRs), as they along with their paired miRNAs target the same gene in the host system (human, cattle), i.e., functionally similar while targeting the host gene. Additionally, the ns-cb-miRs targeting genes in host systems are referred to as functionally non-similar cb-miRs (fns-cb-miRs). To identify the probable targeted genes, the parameters (i) maximum expectation value = 3, (ii) length of complementary score = 20, (iii) maximum energy to unpair target site = 25, and (iv) translation inhibition = 9 nt–11 nt range were set in the psRNATarget Server [23,27,28] (Figure 1). As the mRNAs of cattle are not available in the psRNATarget server, the mRNAs collected from NCBI were uploaded in said server for identifying the genes targeted by s-cb-miRs, following the approach outlined above for humans. However, the targeted genes of fns-cb-miRs were identified from the psRNATarget server by submitting the fns-cb-miRs alone as input for humans and cattle separately.

### 2.4. Functional Annotation and Pathway Analysis of cb-miRs’ Targeted Genes

The human and cattle targeted genes of both fs-cb-miRs (33) and fns-cb-miRs (159) were submitted to ShinyGO v.0.77 [29] and String v.11.5 [30] for functional annotation and KEGG pathway [31] analysis, respectively. The gene ontology (GO) analysis was performed through AgriGO v2.0 tool [32]. Subsequently, the WEGO tool [33] was used for the representation of GO terms in different classes: biological process, cellular component and molecular function. Further, the significant GO terms were filtered out on the basis of *p*-value (<0.05) and FDR (<0.05). The pathway analysis of human and cattle genes targeted by cb-miRs was carried by KEGG mapper [34]. Finally, the gene regulatory network analyses involving the identified cb-miRs and their target genes of humans and cattle were performed by Cytoscape v3.3.0 [35].

### 2.5. Disease Association with cb-miRs’ Target Genes

The targeted genes that were significantly annotated in human were mapped against the DisGeNET database [36] for the analysis of their disease association. The evidence levels of gene–disease association, developed by the NIH-funded Clinical Genome Resource (ClinGen), were qualitatively classified into (i) definitive, (ii) strong, (iii) moderate, (iv) limited, (v) conflicting evidence and (vi) no reported evidence categories [37]. It was reported earlier that the predicted cross-kingdom targeted genes of human have plausible association with several diseases [38]. In case of cattle, the identified targeted genes were searched in literature and CGRIS (http://bioinformatics.iasri.res.in/cgris; accessed on 29 June 2023) for gene–disease association.

## 3. Results

### 3.1. Identification of fs-cb-miRs and fns-cb-miRs to Human and Cattle miRNAs

**Figure 2:**
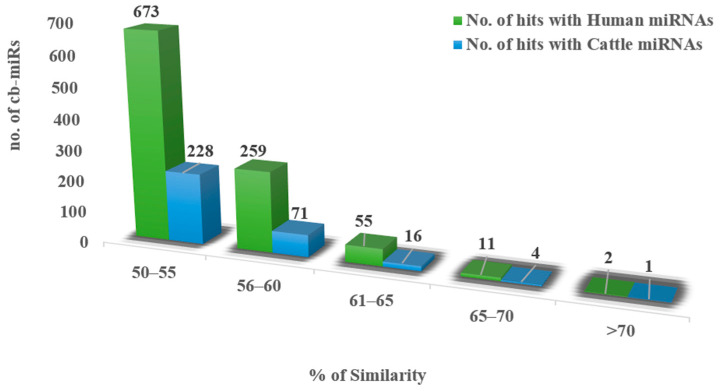
X-axis represents different ranges of % similarities whereas the y-axis represents the number of cb-miRs having similarities with human miRNAs (green color) and cattle miRNAs (blue color).

**Figure 3:**
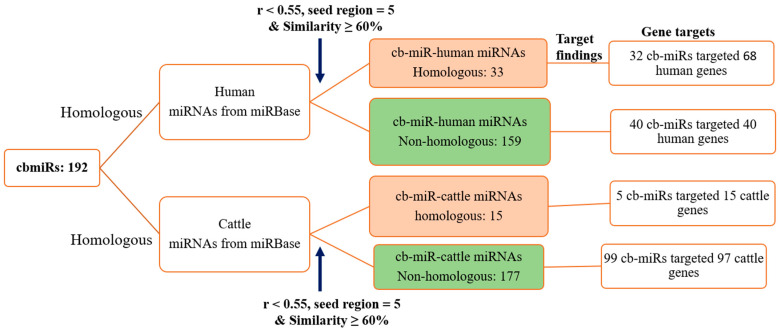
Functional similarity of cluster bean miRNAs (cb-miRs) with human and cattle miRNAs. The observed functionally similar (fs) and functionally non-similar (fns) cb-miRs targeting genes in humans and cattle are indicated in orange color and green color boxes, respectively.

**Figure 4:**
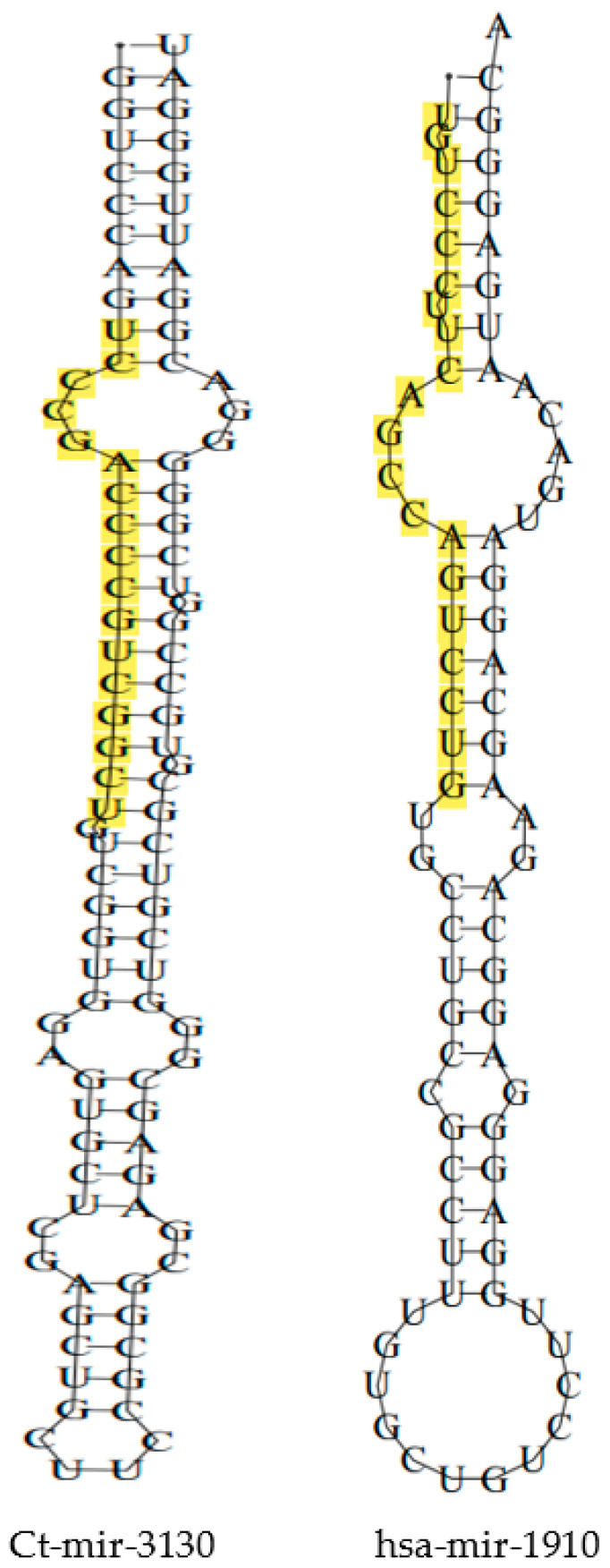
Two-dimensional structures of fs-cb-miR and its corresponding human miRNA with the location of mature miRNA being highlighted.

With input of 192 cb-miRs and 2042 human miRNAs, the similarity hits were found from the MirCompare tool. Similarly, while providing 192 cb-miRs and 206 cattle miRNAs as input to the MirCompare tool. The functional similarities between cluster bean miRNAs and human/cattle miRNAs are given in Figure 2 under different ranges of % similarity. Subsequently, cut off values of parameters r < 0.55, similarity ≥ 60% and seed region = 5 were kept in the MirCompare tool. This has resulted in 33 as fs-cb-miRs and 159 as fns-cb-miRs with human miRNAs, while 15 unique cb-miRs resulted as fs-cb-miRs and 177 as fns-cb-miRs with cattle miRNAs (Figure 3). The predicted secondary structures of both fs-cb-miRs and their corresponding human miRNAs were checked for the location of mature miRNAs. As an example, the secondary structures of Ct-mir-3130 and hsa-mir-1910 are given in Figure 4. The human miRNAs: hsa-miR-6754-3p and hsa-miR-6804-5p were found to have similarity with the cb-miRs: Cte-miR824-3p and Cte-miR6183, respectively. The percentage similarity in the former case was 73%, whereas in the latter case it was 71%. The cattle miRNAs bta-miR-7865 and bta-miR-2338 were found to have similarity with the cb-miRs Ct-miR-3061 and Ct-miR-3033 of cluster beans, respectively. The observed similarities in the former and latter cases were 74% and 69%, respectively.

### 3.2. Prediction of Target Genes of cb-miRs in Human and Cattle

The identified 33 (~17%) and 15 (~7.8%) fs-cb-miRs along with their corresponding paired miRNAs in humans and cattle, respectively, were subjected to the target gene prediction using the psRNATarget server. Subsequently, a total of 32 and 5 fs-cb-miRs have uniquely targeted 68 and 15 genes in human and cattle, respectively (Figure 3). Similarly, we found 40 targeted genes for 40 fns-cb-miRs in case of human and 97 unique targeted genes for 99 fns-cb-miRs in cattle (Figure 3). The perfect and near perfect complementary matches of cb-miRs to their target mRNAs show the probability of post-transcriptional gene expression by mechanisms such as translation inhibition and cleavage of mRNA [39].

### 3.3. Functional Annotation and Pathway Analysis of Human and Cattle Genes Targeted by cb-miRs

**Figure 5:**
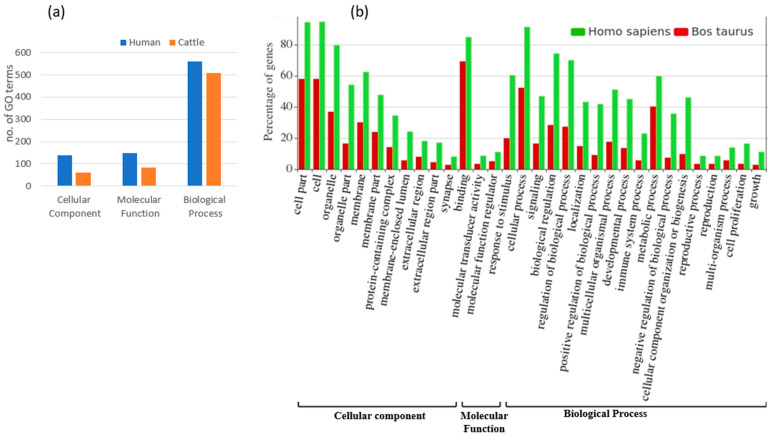
(**a**) Number of GO terms involved in cellular component, molecular function and biological process for targeted genes of fs-cb-miRs in human (blue) and cattle (red). (**b**) Functional annotation and GO terms of targeted genes of fs-cb-miRs in cattle (red) and human (green).

**Figure 6:**
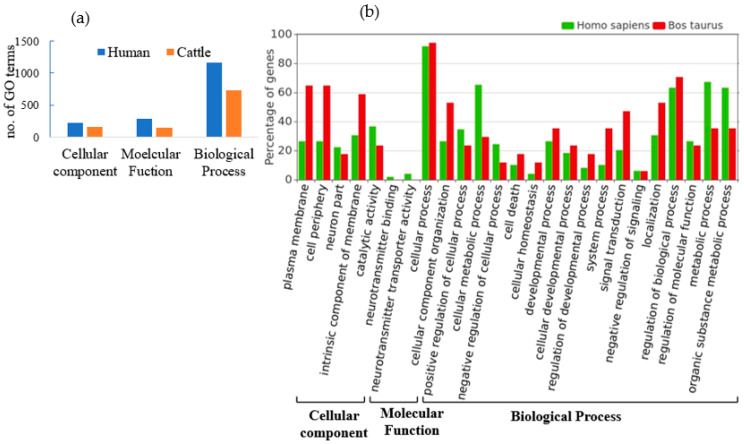
(**a**) Number of GO terms involved in cellular component, molecular function and biological process for human (blue) and cattle (orange) for targeted genes of fns-cb-miRs. (**b**) Functional annotation and GO terms of targeted genes of fns-cb-miRs in cattle (red) and human (green).

A total of 68 and 15 unique genes in humans and cattle, respectively, were identified as targets for fs-cb-miRs from the psRNAtarget server. Subsequently, the ShinyGO v.0.77 and String v.11.5 were used to annotate and identify the involvement of targeted genes in various pathways. The annotation terms were filtered out with *p* value < 0.05 resulting in 846 terms in humans and 653 terms in cattle. Among the GO annotation terms of human, 16.31% (138), 17.38% (147) and 66.31% (561) were found to be involved in cellular component, molecular function and biological process, respectively, for targeted genes of fs-cb-miRs whereas these annotation terms in cattle were 9.34% (61), 12.71% (83) and 77.95% (509) for targeted genes of fs-cb-miRs (Figure 5). A total of 24 and 11 KEGG pathways were identified for targeted genes of fs-cb-miRs in human (Table 1) and cattle (Table 2), respectively. It was also observed in the tables that few genes like HMGCS2, LCP2, PPP2R5C and GABRA6 in humans and genes like ATG2B in cattle have participated in more than five pathways. On the other hand, 40 fns-cb-miRs have targeted 40 human genes and 99 fns-cb-miRs have targeted 97 distinct cattle genes. Among the GO annotation terms of targeted genes of humans for fns-cb-miRs, 13.61% (230), 17.40% (294) and 68.99% (1166) were found to be involved in the cellular component, molecular function and biological processes whereas in the case of cattle the percentage of GO terms were 15.14% (157), 14.08% (146) and 70.78% (734) for targeted genes of fns-cb-miRs (Figure 6). Also, 46 and 44 KEGG pathways were identified for targeted genes of fns-cb-miRs in human (Table 3) and cattle (Table 4), respectively.

### 3.4. Gene Regulatory Network Analysis

**Figure 7:**
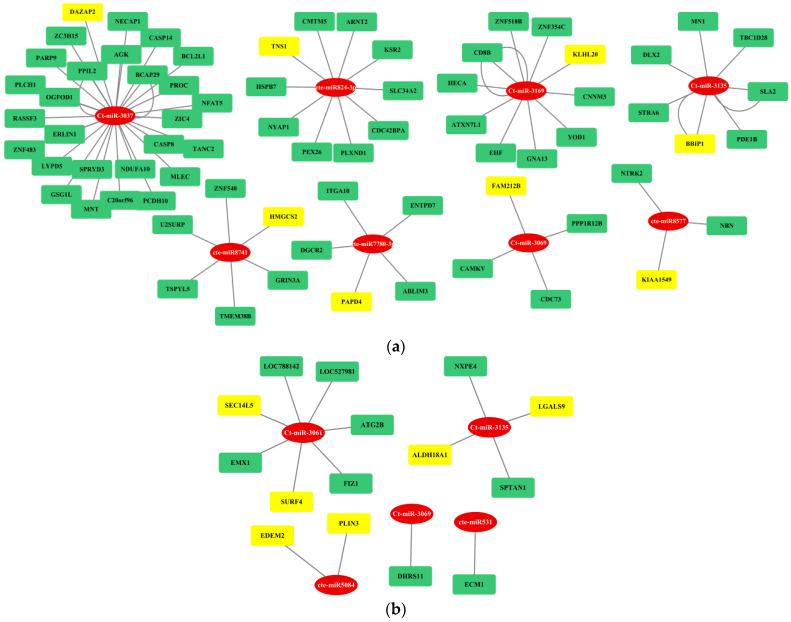
Gene regulatory network analysis of fs-cb-miRs (red color) and their cross-kingdom targeted genes (yellow or green colors) in (**a**) humans and (**b**) cattle. Here, miRNA and mRNA are represented in eclipse and rectangular shapes, respectively. Genes in yellow color show their association with diseases.

**Figure 8:**
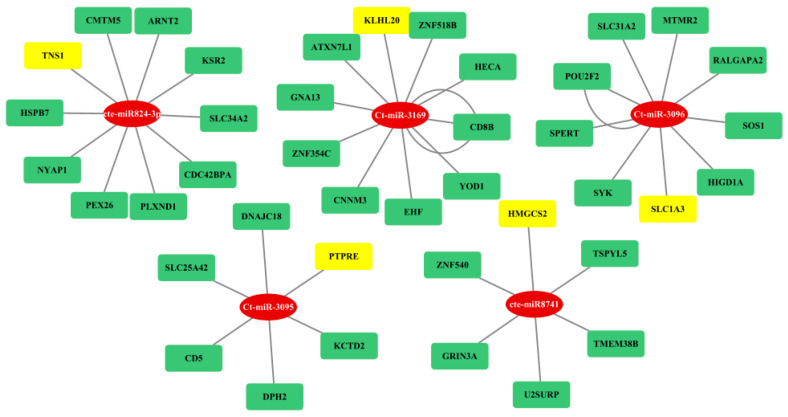
Gene regulatory network analysis of fns-cb-miRs (in red color) and their targeted genes (yellow or green colors) in human. Here, miRNA and mRNA are represented in rectangular shapes (miRNAs in red color and mRNAs in both green and yellow colors). Rectangular boxes in yellow color show the genes associated with diseases.

The regulatory network between fs-cb-miRs and their targeted genes in humans and cattle were visualized by cytoscape and depicted in Figure 7. The integrative analyses of cb-miRs and their target genes responsible for diseases in cattle and human provide useful information to understand complex biological systems and processes involved in miRNA–mRNA–disease associations. A total of eight and five cb-miRs (ellipse shape) showed their likely integration with the gene networks in humans and cattle, respectively. The targeted genes in the network are shown in rectangular boxes (Figure 7a,b). The length of edges in the network shows the strength of unpaired energy (UPE) that in turn depicts the interaction between miRNA and mRNA. The fs-cb-miRs like Ct-miR-3037, Ct-miR-3169, Cte-miR824-3p, Ct-miR-3135, Cte-miR8741, and Cte-miR7780-3p were found to target more than four genes. The targeted genes of fs-cb-miRs, viz., DAZAP2, KLH20, TNS1, BBIP1, HMGCS2, PAPD4, FAM212B and KIAA1549 are having disease associations. Similarly, in the case of cattle, fs-cb-miRs like Cte-miR5084, Cte-miR531, Ct-miR-3061, Ct-miR-3069 and Ct-miR-3135 have targeted the genes having several gene associations. The genes having disease associations are highlighted in yellow color. Further, a regulatory network was developed for targeted genes of fns-cb-miRs and it was observed that few genes are participating in the gene regulatory network in the case of humans (Figure 8) while no regulatory network was formed in the case of cattle.

### 3.5. Association of Target Genes of fs-cb-miRs and fns-cb-miRs with Human and Cattle Diseases

The targeted genes of fs-cb-miRs in human were found to be associated with multiple diseases given in DisGeNET database, which is one of the largest available collections of genes and variants involved in human diseases. A total of eight out forty targeted genes were found to be associated with diseases based on first four evidence levels of DisGeNET. Most of the gene–disease associations were categorized into “strong” (50%) evidence level and others into “definitive” (3%), “moderate” (8%) and “limited” (39%). The fs-cb-miRs, targeted genes and their associated diseases, disease association type, and references are given in Table 5. Further, it was observed that the fs-cb-miR-targeted gene KLHL20 was involved in Alzheimer’s disease (Figure 7a) while another gene, PAPD4, was found as a biomarker for diseases Hepatitis C virus infection. Additionally, the variations in the targeted genes: TNS1, FAM212B and KIAA1549 have led to the diseases such as malignant tumors of the breast, Crohn’s disease and Pilocytic astrocytoma, respectively (Table 5). In contrast, the fns-cb-miRs and their targeted gene-association information along with references are given in Table 6. Here, the targeted genes like DNTT, PTPRE, CNRIP1, CLEC4G, LMTK2, PHKA1, and KLHL were found associated with diseases like Schrizophrenia, Asthma, Colorectal Carcinoma, Liver Carcinoma, Glycogen Storage Disease, Chronic Lymphocytic Leukemia, Alcoholic Intoxication and Alzheimer diseases with a disease association type as a biomarker (Table 6).

In the case of cattle, the literature was searched for analyzing the gene–disease association. The fs-cb-miR targeted gene–disease association information and corresponding references are given in Table 7. A total of 11 out of 15 targeted genes, PLIN3, EDEM2, ECM1, SURF4, SEC14L5, DHRS11, LGALS9, ALDH18A1, SPTAN1, NXPE4 and FIZ1 of fs-cb-miRs, were found associated with the diseases like bovine respiratory disease, mastitis resistance, and Hyperprolinemia type II, etc. (Table 7). On the other hand, the fns-cb-miRs and their targeted gene–disease association information are given in Table 8 the genes like CFLAR, TRPA1, RB1 and SCNN1B were found involved in malignant glioma, respiratory disease, sporadic retinoblastoma and renal disease, respectively, in cattle (Table 8).

## 4. Discussion

The cross-kingdom role of edible plant miRNAs, also known as food-derived miRNAs, play an important role in inter-species regulation [3,38,80,81,82,83]. These studies further demonstrated that the plant miRNAs act in a similar manner to human miRNAs after entering the gastrointestinal (GI) tract [82]. The stability of plant-derived miRNAs has been studied under high temperature and chemical degradation processes as well as in the gastrointestinal tract of humans and animal serum [84,85]. The small-molecule carriers such as exosomes, microvesicles and high-density lipoprotein are responsible for the stability of exogenous miRNAs as they protect from degradation [5,86,87]. The miRNA-mir2911 of Chinese herb honeysuckle (*Lonicera japonica*) is highly stable and helps in protecting against influenza virus (Zhou et al., 2015) and novel Coronavirus SARS-CoV-2 [88]. Similarly, “miR471” and “miR519” from lettuce targeted the Hepatitis B virus (HBV) [81]. Recently, it was reported that plant miRNA “miR159” helps in reducing the proliferation of breast cancer cells [39]. Moreover, the plant derived miRNAs, which regulate multiple gene expression in cross-kingdom species, may lead to a new approach to cast light on the nutritional and functional value of plants [18]. It was suggested that exogenous miRNAs (plant miRNAs) have targeted the genes in the human genome [6,89]. Similarly, the miRNAs of *Moringa oleifera*, *Ocimum basilicum* and *Medicago truncatula* target the genes in human [26,90,91]. Likewise, the role of plant-derived miRNAs has been studied in mammals including humans and mice in the recent past. To be specific, the understanding of plant-derived miRNAs in mammals encompassing antiviral, antitumor, anti-inflammatory, anti-apoptotic, immune-modulating, and regulatory impacts on intestinal function were studied recently [82]. It has also been studied that the MiR171 variant from the Arabidopsis and tomato targeted the mTOR pathway of the HEK293 cell of humans [92]. Interestingly, the human miRNAs “hsa-miR-21-5p” and “hsa-miR-24-3p” targeted the gene cyclin-dependent kinase inhibitor Sol1 of Candida albicans and inhibit its cell growth [93]. These findings support the cross-kingdom analysis. However, to our limited knowledge, the role of plant-derived-miRNAs in cattle has been reported for the first time by us.

The present study is carried out to (i) identify functionally similar-cb-miRs (fs-cb-miRs), i.e., cbmiRs having sequence similarities with human and cattle miRNAs as well as satisfying structural properties of miRNAs and target the same gene as that targeted by their corresponding similarity pairs in host organisms (human, cattle), (ii) identify functionally-non-similar-miRNAs (fns-cb-miRs) that regulate genes in the host (human, cattle) organisms, and (iii) the involvement of targeted genes in regulatory networks, pathways and disease association. The fs-cb-miRs were studied for functional and probable evolutionary importance in the host systems like humans and cattle. Whereas fns-cb-miRs were studied for their functional role in humans and cattle. The targeted genes of both fs-cb-miRs and fns-cb-miRs in host systems have also been studied through regulatory network and pathway analysis. In addition, the disease associations of the targeted genes have been studied.

Our results revealed high similarity to the extent of more than 70% between human miRNA “hsa-mir-6754-3p” and cb-miR “Cte-mir824-3p” as well as between “hsa-mir-6804-5p” and “Cte-mir6183”. The hsa-mir-6754-3p and hsa-mir-6804-5p target the genes TNS1 and FNDC3A, respectively. These targeted genes code for the corresponding transcription factors that are responsible for the regulation of gene expression [42]. Similar findings have been reported that 84 miRNAs of wheat have targeted the 787 human genes [94]. Our findings on fs-cb-miRs fall in line with the results reported earlier [95] about the similarity between the human miRNA “hsa-mir341” and olive plant miRNAs:oeu-sR20, oeu-sR27, oeu-sR34.

In the case of cattle, the fs-cb-miR “Ct-mir-3061” has 74% similarity with the cattle miRNA “bta-miR-7895” and was observed to interact with the FIZ1 gene responsible for regulating cellular processes as well as being associated with various biological functions [74]. Whereas the fs-cb-miR “Ct-miR-3033” has 69% similarity with the cattle miRNA “bta-mir-2338” and was found interacting with a GAIP-interacting protein C-terminus (GIPC3) gene that has a potential role in cell growth, differentiation, and survival [75].

The gene regulatory networks play an important role in various vital processes of life including cell differentiation, metabolism, cell cycle and signal transduction [95]. The fs-cb-miR “Cte-miR824-3p”, which is functionally similar to the human miRNAs hsa-miR-4279, hsa-miR-6754-3p, hsa-miR-6845-3p, hsa-miR-6887-3p, hsa-miR-877-3p and hsa-miR-6894-5p, was found to interact with the human genes TNS1, SLC34A2, KSR2, NYAP1, HSPB7, CMTM5, PLXND1, ARNT2, and CDC42BPA involved in regulation of phosphate homeostasis [96], cell growth and differentiation [97], neuronal development and synaptic plasticity [98], maintaining cardiac structure and function [99], tumor suppression [100], angiogenesis during development [101], neuronal development [102], and cytoskeletal organization [103] and cancer [104], respectively. Similar findings on the regulation of genes by miRNAs have also been reported in *Bacopa monnieri* [105] and wheat [94].

The KEGG pathway analysis of the targeted genes of fs-cb-miRs in both humans and cattle revealed that the targeted genes were involved in multiple pathways (Table 1). For example, the targeted gene LCP2 is involved in eight pathways including important pathways like, Rap1 signaling pathway, osteoclast differentiation, Yersinia infection, platelet activation, T-cell receptor signaling pathway, and Fc epsilon RI signaling pathway [51]. Whereas in the case of cattle, only two genes ALDH18A1 and ATG2B were involved in more than one pathway [31].

Further, we investigated the gene–disease associations in human and cattle by using cb-miRs targeted genes through DisGeNET database [36] and literature, respectively. Here, 17 targeted genes of fs-cb-miRs show their involvement in 16 different diseases in human. Few targeted genes of fs-cb-miRs like TNS1 (Cte-miR824-3p), UBE2K (Cte-miR168), FNDC3A (Cte-miR6183) and PAPD4 (Cte-miR7780-3p) were involved in malignant tumors of the breast, Alzheimer’s disease, Angelman syndrome and Hepatitis C virus infection, respectively. The gene–disease associations (Table 5) were reported earlier [42,45]. These findings are in line with earlier reported findings such as the miRNAs “pku-miR167a-5p” and “pku-miR167b-3p” from *Picrorhiza kurroa* having targeted the genes:“PPP3R2” and “MYOZ3”, respectively, involved in astheno-zoospermia and muscular dystrophy in humans [106]. In the case of cattle, it was observed that six cb-miRs have targeted twelve genes. The targeted genes, PLIN3, EDEM2, SURF4, and LGALS9, with mutations/variations have led to metabolic diseases, bovine osteoporosis, mastitis resistance and bovine respiratory disease, respectively. These mentioned gene–disease associations were reported earlier in the literature [64,65,67,70]. Interestingly, lncRNA “LOC788142” was targeted by the fs-cb-miRs and found expressed in various tissues in cattle, including muscle, liver, and adipose tissue [107].

Our findings also revealed that fns-cb-miRs target the human genes HMGCS2, PPP2R5C, LCP2, and GABRA6 involved in more than four pathways such as signaling pathways, metabolism regulation pathways and a few disease related pathways. The signaling pathways are calcium, Glucagon, insulin, AMPK, and PI3k-Aktin. Further, the gene regulatory network was developed between the targeted genes and fns-cb-miRs. The gene–disease association study revealed that 22 targeted genes were associated with 17 different diseases. Here, mostly genes are working as biomarkers in gene–disease-associations. Three genes, namely, CLEC4G, LCP2, ST8SIA1 are acting as biomarkers in liver carcinoma [51]. Similarly, gene DNTT and UHMk1 are predicted as biomarkers in Schizophrenia. The targeted genes of fns-cb-mirs of cattle were also studied and we found that nine targeted genes are involved in forty-four pathways. These pathways were mainly related to signaling, but few disease-related pathways were also found. Furthermore, four fns-cb-miRs and their respective targeted genes were associated with several diseases in cattle. Here, dysfunction in the fns-cb-miR’s targeted gene SCNN1B lead to renal disease in cattle [79]. Thus, the above findings may pave the way for insights into the role of plant-derived miRNAs in animal cells. However, wet-lab validation of findings is required for a deeper understanding and confirmation of the role of plant-derived miRNAs across the animal kingdom.

## 5. Conclusions

The plant-derived miRNAs are likely to have an expected functional similarity with the miRNAs of cross-kingdom species like humans and cattle due to the use of plant parts as their dietary intake. In the present study, we found fs-cb-miRs and fns-cb-miRs having functional similarity and functional non-similarity with the miRNAs of humans and cattle through an in silico approach. These fs-cb-miRs were found to target various genes like LCP2, GABRA6, and MYH14 in humans and ATG2B and DHRS11 in cattle. Mostly the fs-cb-miRs’ targeted genes of humans were involved in the signaling pathways like PPAR, Rap1, T cell receptor, Fc epsilon RI, and Retrograde endocannabinoid. The gene–disease-association in humans showed that targeted genes of fs-cb-miRs like KLHL20, BBIP1, and PAPD4 have acted as biomarkers for the diseases such as Alzheimer’s disease, Bardet-Biedl syndrome 18, and Hepatitis C virus infection, respectively. In cattle, the targeted genes of fs-cb-miRs are involved in Apoptosis, Necroptosis, Chagas disease and Hepatitis C. Further, the gene–disease-association showed that mutation/variation in the genes like EDEM2, ECM1, SURF4, and DHRS11 caused bovine osteoporosis, bovine hereditary angioneurotic edema, mastitis resistance and bovine viral diarrhea in cattle. In contrast, fns-cb-miRs’ targeted genes in humans and cattle also showed their involvement in various pathways and diseases. Genes like NRM, SLC6A6, and PTPRE are involved in Rheumatoid Arthritis, Myocardial Ischemia and Asthma diseases in humans. In the case of cattle, genes like TRPA1, RB1 and SCNN1B are predicted to be involved in respiratory diseases, retinoblastoma and renal diseases, respectively. Thus, our findings reflect the translation of plant/food derived miRNAs in cross kingdom species and their functional significance in the gene–disease associations. In the future, these plant-derived miRNAs may become an alternative to current trend of using synthetic-miRNA-based drugs, which are time-consuming and expensive compared to the natural miRNA supplements.

## Figures and Tables

**Table 1:** List of human genes targeted by fs-cb-miRs and their involvement in KEGG pathways.

Targeted Gene	KEGG Id	Pathway
HMGCS2	hsa00072	Synthesis and degradation of ketone bodies
	hsa00280	Valine, leucine and isoleucine degradation
	hsa00650	Butanoate metabolism
	hsa00900	Terpenoid backbone biosynthesis
	hsa01100	Metabolic pathways
	hsa03320	PPAR signaling pathway
UBE2K	hsa04120	Ubiquitin mediated proteolysis
MYH14	hsa04270	Vascular smooth muscle contraction
	hsa05130	Pathogenic Escherichia coli infection
	hsa04530	Tight junction
LCP2	hsa04810	Regulation of actin cytoskeleton
	hsa04015	Rap1 signaling pathway
	hsa04380	Osteoclast differentiation
	hsa05135	Yersinia infection
	hsa04611	Platelet activation
	hsa04650	Natural killer cell mediated cytotoxicity
	hsa04660	T cell receptor signaling pathway
	hsa04664	Fc epsilon RI signaling pathway
GABRA6	hsa04723	Retrograde endocannabinoid signaling
	hsa04727	GABAergic synapse
	hsa04742	Taste transduction
	hsa05032	Morphine addiction
	hsa05033	Nicotine addiction
	hsa04080	Neuroactive ligand-receptor interaction

**Table 2:** List of cattle genes targeted by fs-cb-miRs and their involvement in KEGG pathways.

Targeted Gene	KEGG Id	Pathway
ALDH18A1	bta00330	Arginine and proline metabolism
	bta01100	Metabolic pathways
	bta01230	Biosynthesis of amino acids
ATG2B	bta04136	Autophagy—other
	bta04140	Autophagy—animal
	bta05010	Alzheimer disease
	bta05014	Amyotrophic lateral sclerosis
	bta05016	Huntington disease
	bta05017	Spinocerebellar ataxia
DHRS11	bta00140	Steroid hormone biosynthesis
EDEM2	bta04141	Protein processing in endoplasmic reticulum
SPTAN1	bta04210	Apoptosis

**Table 3:** List of human genes targeted by fns-cb-miRs and their involvement in KEGG pathways.

Targeted Gene	KEGG Id	Pathway
ABI2	hsa04810	Regulation of actin cytoskeleton
COL22A1	hsa04974	Protein digestion and absorption
DNTT	hsa04640	Hematopoietic cell lineage
	hsa03450	Non-homologous end-joining
FLI1	hsa05202	Transcriptional misregulation in cancer
FMO3	hsa00982	Drug metabolism—cytochrome P450
GABRA6	hsa04727	GABAergic synapse
	hsa05032	Morphine addiction
	hsa04080	Neuroactive ligand-receptor interaction
	hsa05033	Nicotine addiction
	hsa04723	Retrograde endocannabinoid signaling
	hsa04742	Taste transduction
HMGCS2	hsa00650	Butanoate metabolism
	hsa01100	Metabolic pathways
	hsa03320	PPAR signaling pathway
	hsa00072	Synthesis and degradation of ketone bodies
	hsa00900	Terpenoid backbone biosynthesis
	hsa00280	Valine, leucine and isoleucine degradation
LCP2	hsa04664	Fc epsilon RI signaling pathway
	hsa04650	Natural killer cell mediated cytotoxicity
	hsa04380	Osteoclast differentiation
	hsa04611	Platelet activation
	hsa04015	Rap1 signaling pathway
	hsa04660	T cell receptor signaling pathway
	hsa05135	Yersinia infection
MYH14	hsa05130	Pathogenic Escherichia coli infection
	hsa04810	Regulation of actin cytoskeleton
	hsa04530	Tight junction
	hsa04270	Vascular smooth muscle contraction
PDSS2	hsa00900	Terpenoid backbone biosynthesis
PHKA1	hsa04(020,922,910)	signaling pathway (Calcium, Glucagon, Insulin)
PPP2R5C	hsa04261	Adrenergic signaling in cardiomyocytes
	hsa04152	AMPK signaling pathway
	hsa04728	Dopaminergic synapse
	hsa05165	Human papillomavirus infection
	hsa03015	mRNA surveillance pathway
	hsa04114	Oocyte meiosis
	hsa04151	PI3K-Akt signaling pathway
	hsa04071	Sphingolipid signaling pathway
SLC1A3	hsa04724	Glutamatergic synapse
	hsa05016	Huntington disease
	hsa04721	Synaptic vesicle cycle
SLC36A4	hsa04974	Protein digestion and absorption
ST8SIA1	hsa00604	Glycosphingolipid biosynthesis
	hsa01100	Metabolic pathways
UBE2K	hsa04120	Ubiquitin mediated proteolysis

**Table 4:** List of cattle genes targeted by fns-cb-miRs and involvement in KEGG pathways.

Targeted Gene	KEGG Id	Pathway
RB1	bta01522, bta04110, bta0421 bta04934, bta05160, bta0516, bta05200	Endocrine resistance, Cell cycle, Cellular senescence, Cushing syndrome, Hepatitis B, Hepatitis C, Pathways in cancer
SCNN1B	bta04742, bta04960	Taste transduction, Aldosterone-regulated sodium reabsorption
CFLAR	bta04064, bta04140, bta04210 bta04217, bta04668, bta05142, bta05160	NF-kappa B signaling pathway, Autophagy—animal, Apoptosis, Necroptosis, TNF signaling pathway, Chagas disease, Hepatitis C
COL4A4	bta04933, bta05146, bta04512, bta04510, bta05200, bta04151, bta04974, bta04926	AGE-RAGE signaling pathway in diabetic complications, Amoebiasis, ECM-receptor interaction, Focal adhesion, Pathways in cancer, PI3K-Akt signaling pathway Protein digestion and absorption, Relaxin signaling pathway,
TRPA1	bta04750	Inflammatory mediator regulation of TRP channels
FAT4	bta04392	Hippo signaling pathway—multiple species
ITGB7	bta05412, bta04514, bta05414, bta05410, bta04672, bta04810, bta05202	Arrhythmogenic right ventricular cardiomyopathy, Cell adhesion molecules, Dilated cardiomyopathy, Hypertrophic cardiomyopathy, Intestinal immune network for IgA production, Regulation of actin cytoskeleton, Transcriptional misregulation in cancer
PIP5K1A	bta05231, bta04144, bta04666, bta00562, bta01100, bta04070, bta04072, bta05135	Choline metabolism in cancer, Endocytosis, Fc gama R-mediated phagocytosis, Inositol phosphate metabolism, Metabolic pathways, Phosphatidylinositol signaling system, Phospholipase D signaling pathway, Yersinia infection
LOC533983	bta04740, bta04975, bta00561	Olfactory transduction, Fat digestion and absorption, Glycerolipid metabolism

**Table 5:** List of fs-cb-miRs targeted gene–disease association in human and their involvement in various disease.

cb-miR Id	Targeted Gene	Association Type	Disease	References
Ct-miR-3037	DAZAP2	Posttranslational Modification	Multiple myeloma	[40]
Ct-miR-3169	KLHL20	Biomarker	Alzheimer’s disease	[41]
Cte-miR824-3p	TNS1	Genetic Variation	Malignant tumor of breast	[42]
Ct-miR-3135	BBIP1	Biomarker	Bardet-Biedl syndrome 18	[43]
Cte-miR8741	HMGCS2	Biomarker	3-hydroxy-3-methylglutaryl-CoA synthase deficiency	[44]
Cte-miR7780-3p	PAPD4	Biomarker	Hepatitis C virus infection	[45]
Ct-miR-3069	FAM212B	Genetic Variation	Crohn’s disease	[46]
Cte-miR8577	KIAA1549	Genetic Variation	Pilocytic astrocytoma	[47]

**Table 6:** List of fns-cb-miRs targeted gene–disease association in human and their involvement in various disease.

Cb-miR Id	Targeted Gene	Association Type	Disease	References
Ct-miR-3034, 3061	DNTT, UHMK1	Biomarker	Schizophrenia	[48]
Ct-miR-3095	PTPRE	Biomarker	Asthma	[49]
Cte-miR5084 cte-miR04	CNRIP1, SLC36A4	Biomarker	Colorectal Carcinoma	[50]
Ct-miR-3035, 3094 Cte-miR8713	CLEC4G, LCP2, ST8SIA1	Biomarker	Liver carcinoma	[51]
Cte-miR5644, 824-3p	NLRC5, TNS1	Altered Expression	Malignant neoplasm	[52]
Ct-miR-3007	NRM	Genetic Variation	Rheumatoid Arthritis	[53]
Cte-miR8577	KIAA1549	Genetic Variation	Pilocytic Astrocytoma	[47]
Cte-miR1134	LMTK2	Biomarker	Malignant neoplasm of prostate	[54]
Cte-miR531	SLC6A6	Biomarker	Myocardial Ischemia	[55]
Ct-miR-3104	TYW1	Genetic Variation	Lymphocyte Count measurement	[56]
Cte-miR117	SUZ12	Genetic Variation	Endometrial Stromal Sarcoma	[57]
Ct-miR-3096	SLC1A3	Causal Mutation	Episodic Ataxia, TYPE 6	[58]
Ct-miR-3041	PHKA1	Genetic Variation	Glycogen Storage Disease, Type IXD	[59]
Ct-miR-3027	SHOX	Genetic Variation	Leri-Weill dyschondrosteosis	[60]
Ct-miR-3015	PPP2R5C	Biomarker	Neoplasm	[61]
Ct-miR-3139	PDSS2	Causal Mutation	Coenzyme Q10 Deficiency	[44]
Cte-miR168	UBE2K	Genetic Variation	Angelman Syndrome	[62]
Cte-miR8741	HMGCS2	Genetic Variation	3-Hydroxy-3-Methylglutaryl-CoA Synthase 2 Deficiency	[44]
Ct-miR-3097	GABRA6	Biomarker	Alcoholic Intoxication, Chronic	[63]
Ct-miR-3169	KLHL20	Biomarker	Alzheimer’s Disease	[41]

**Table 7:** List of fs-cb-miR targeted genes in cattle, disease associations and references.

Cb-miR Id	Gene	Description	References
Cte-miR5084	PLIN3	Associated with various metabolic diseases, primarily expressed in adipose tissue.	[64]
	EDEM2	Mutations leads genetic disorder, bovine osteoporosis.	[65]
Cte-miR531	ECM1	Mutations leads genetic disorder, bovine hereditary angioneurotic edema (HANE).	[66]
Ct-miR-3061	SURF4	In relation to milk production traits and mastitis resistance.	[67]
	SEC14L5	Variations/mutation leads susceptibility to mastitis, lead to reduced milk production and quality and involved in regulating immune function in response to bacterial infection.	[68]
Ct-miR-3069	DHRS11	Variations/mutation lead susceptibility to infectious diseases like bovine viral diarrhea virus in cattle.	[69]
Ct-miR-3135	LGALS9	Expressed in bovine respiratory disease (BRD, and may be involved in the development and progression of BRD.	[70]
	ALDH18A1	Mutations lead Hyperprolinemia type II, rare autosomal recessive disorder.	[71]
	SPTAN1	Mutations leads Cerebellar abiotrophy, neurological disorder that affects the cerebellum.	[72]
	NXPE4	SNP (single nucleotide polymorphism) in the NXPE4 gene was significantly associated with milk yield and fat content in milk.	[73]
	FIZ1	Variations lead BRD is a multifactorial disease.	[74]
Ct-miR3033	GIPC3	Potential role in cell growth, differentiation and survival.	[75]

**Table 8:** List of fns-cb-miRs and their targeted genes of cattle and involvement in different disease.

cb-miR Id	Gene	Description	References
Cte-miR824-3p	CFLAR	Malignant glioma, cancer	[76]
Cte-miR5644	TRPA1	Respiratory diseases	[77]
Ct-miR-3017	RB1	Mutations lead sporadic cases of retinoblastoma	[78]
Ct-miR-3145	SCNN1B	Dysfunctions in SCNN1B renal diseases	[79]

## Data Availability

The data presented in this study are openly available either in literature or in openly available repositories (public domain databases). The miRNAs of cluster bean are available in literature [22,23]. The miRNAs of cattle and human are available in miRbase ([24]; https://mirbase.org/download/hairpin.fa, accessed on 29 June 2023). The mRNA sequences of cattle are available in National Center for Biotechnology Information (NCBI) at https://www.ncbi.nlm.nih.gov/, accessed on 29 June 2023, whereas human mRNAs are available in the database of psRNATarget server ([25]; https://www.zhaolab.org/psRNATarget/, accessed on 29 June 2023].
